# Anxiety in the Elderly Can be a Vestibular Problem

**DOI:** 10.3389/fpubh.2015.00216

**Published:** 2015-09-24

**Authors:** Eli Carmeli

**Affiliations:** ^1^University of Haifa, Haifa, Israel

**Keywords:** elderly, anxiety, vestibular system, dizziness, vertigo

Anxiety and vestibular disorders appear to be intimately related. Anxiety can be a symptom of underlying health problems, such as dizziness, and *vice versa*. Both sets of symptoms can deprive patients of social interactions, social encounters, and participation in daily activities.

Psychological disorders, such as anxiety and depression, on one hand, and vestibular disorders, such as dizziness or vertigo, on the other, are fairly common complaints among elderly people, and both have a long list of possible underlying causes. Anxiety and dizziness are non-specific complaints. Individuals with anxiety may have impaired sleep patterns, nightmares/bad dreams, increased motor tension, irritability, restlessness, uneasiness, and thoughts, such as fear of dying, whereas dizziness may cause lightheadedness, unsteadiness, feeling faint, or visual disturbances.

Dizziness and anxiety are common complaints, but at the same time may reflect health problems, such as cardiovascular disorders, hyperventilation, orthostatic hypotension, orthostasis, and drug side effects. Thus, establishing a valid or differential diagnosis is indispensible in view of the range of possible causes and to allow effective management.

The vestibular system is a sensory system that can begin to function poorly with age, which may cause falls and eventually lead to serious clinical problems and a diminished quality of life. The prevalence estimates of vestibular vertigo with aging range from 5.0 to 8.0% (1). Vestibular problems, such as vertigo or benign paroxysmal positional vertigo (BPPV), are common in older adults, causing dizziness, and poor postural stability (2). Yet, such symptoms should not necessarily or immediately be attributed solely to aging processes of the inner ear. They could be associated with primary psychological disturbances, such as anxiety (3).

Elderly people often suffer from behavioral abnormalities or psychological disturbances, such as anxiety, stress, panic, and depression, which may accompany dizziness, feeling faint, unsteady or light-headed, or experiencing a swaying or spinning sensation (i.e., vertigo) (4–6). Prevalence estimates of anxiety disorders in older ages range from 3.0 to 14.0%. Anxiety is a psychological problem that sometimes should be considered as an adjunct problem to vestibular system impairments, such as dizziness and vertigo. Yet, the pathway explaining the association between anxiety and vestibular disorder is complex. These two symptoms – anxiety and dizziness – share some neural pathways, which may help explain why dizziness occurs among many patients suffering from anxiety (7–9). Nevertheless, psycho-geriatric physicians can play a critical role in addressing the concerns and needs due to a vestibular disorder, by diagnosing and treating the psychological as well as the physical problems of older adults.

Three hypotheses might explain the pathways describing the association between anxiety and a vestibular disorder, such as dizziness, among older adults. The first is a psychogenic vestibular disturbance among patients who have normal vestibular tests and laboratory evaluations. For example, in unilateral Ménière’s disease, recurrent vestibulopathy and psychogenic vertigo were found to be a specific vestibular disturbance (10).

The second is somatopsychic impairment of those who exhibit a primary inner ear disturbance causing anxiety (11). In other words, a vicious cycle exists, where signals from the inner ear are misinterpreted by the central nervous system and signify an immediate threat, which increases anxiety. A case report and literature review supported this hypotheses and showed that increased anxiety amplifies misinterpretation (11), and the disturbances is even worse when the patient adopts this bad habituation, which makes dizziness along with anxiety a constant, coexisting symptom.

The third hypothesis is that of a psycho-cognitive problem (12). Many elderly people complain of difficulty multitasking, meaning that their balance control is decreased due to a diversion of attention. This results in less mental availability for psychological processing and therefore causes anxiety. An open trial of mindfulness-based cognitive therapy program that focused on intensive training in mindfulness meditation and integrated principles of cognitive behavior therapy found to be an acceptable and effective treatment for patients suffering from anxiety disorder (13).

Too often, family physicians and even geriatric physicians do not attribute symptoms of anxiety to an “organic” problem or to a vestibular explanation. This situation frequently causes uncertainty between the “lack of an organic diagnosis” or a psycho-somatic problem – “it is all in your head.” In other words, patients with anxiety should not be labeled with a mental health problem unless other diagnoses have been ruled out.

Therefore, the primary aim of evaluation of a patient with dizziness versus anxiety is a thorough discussion and explanation of the problem and its clinical appearance. This should be followed by a careful physical examination in order to assess possible underlying causes; for example, cardiovascular disease (e.g., postural hypotension, carotid sinus syndrome, aortic stenosis, subclavian steal syndrome, cardiac arrhythmias), neurological disease (e.g., multiple sclerosis, Parkinsonism, brain tumor); metabolic disease (e.g., hypoglycemia, hypothyroidism), or an adverse drug reaction (e.g., due to anti-emetics or calcium-channel antagonists).

Management of anxiety or a vestibular disorder has some common components. They both require the patient to undertake an exercise regimen and to take responsibility to ameliorate the symptom or remediate the problem. Treating dizziness with a management-oriented approach should include non-traditional strategies, such as referral to psychiatry, and *vice versa*. Cognitive behavioral therapy (CBT) should also be incorporated into vestibular rehabilitative regimens. When anxiety treatment succeeds it often results in decreased dizziness and increased activity and socialization.

In conclusion, with elderly patients, the family physician may not know the cause of the anxiety or dizziness. In other words, how do family physicians know that an arbitrary combination of sensory, central, and motor deficits is an adequate explanation for anxiety or for a vestibular disorder.

Many patients have dizziness associated with or attributed to anxiety. Therefore, the family physician must be able to distinguish between psychogenic and vestibular disorders. It is not unusual to make a wrong diagnosis regarding why a person is anxious or dizzy, even after a very thorough evaluation. One should remember that anxiety is sometimes related to a vestibular disorder and *vice versa*, in a very complex way. Each may be a cause, as well as a consequence of the other.

## Conflict of Interest Statement

The author declares that the research was conducted in the absence of any commercial or financial relationships that could be construed as a potential conflict of interest.
